# A network analysis of benefit finding, posttraumatic growth, anxiety, and depression in breast cancer patients

**DOI:** 10.3389/fonc.2026.1845985

**Published:** 2026-09-17

**Authors:** Yuqing Wang, Yan Zhu, Wei Wang, Keying Guo, Weina Du, Ling Cheng, He Gao, Zhongtao Zhou, Jing Zhang

**Affiliations:** 1 College of Nursing, Bengbu Medical University, Bengbu, Anhui, China; 2 College of Mental Health, Bengbu Medical University, Bengbu, Anhui, China

**Keywords:** anxiety, benefit finding, breast cancer patients, depression, network analysis, posttraumatic growth

## Abstract

**Purpose:**

Breast cancer patients often experience psychological distress, such as anxiety and depression, during diagnosis and treatment; however, positive psychological changes, including benefit finding (BF) and posttraumatic growth (PTG), may also occur. This study aimed to construct a co-occurrence network of BF, PTG, anxiety, and depression and to identify central and bridge dimensions across different psychological communities.

**Methods:**

A total of 707 breast cancer patients were recruited from five tertiary hospitals in northern Anhui Province, China, between September 2025 and January 2026. Participants completed the Benefit Finding Scale (BFS), the Posttraumatic Growth Inventory (PTGI), and the Hospital Anxiety and Depression Scale (HADS). Network analysis was conducted to construct the network, identify central and bridge dimensions, and assess network stability. Expected influence (EI) and bridge expected influence (BEI) were calculated to assess centrality and bridge centrality, respectively.

**Results:**

A total of 707 breast cancer patients participated in the study. Network analysis revealed that BFS4 (“personal growth”, EI = 1.034) was the most central dimension in the co-occurrence network. The strongest edge was observed between HADS1 and HADS2 (“anxiety–depression”, edge weight = 0.527), both within the distress community. Key bridge dimensions included BFS4 (“personal growth”, BEI = 0.504), PTGI4 (“relating to others”, BEI = 0.481), and BFS3 (“worldview”, BEI = 0.401). The network showed high stability.

**Conclusions:**

“Personal growth” (BFS4) occupied both central and bridge positions in the estimated network. Together with the bridge positions of PTGI4 (“relating to others”) and BFS3 (“worldview”), these findings highlight the potential relevance of personal growth-related resources, interpersonal relationships, and worldview when considering psychological distress and positive psychological constructs in breast cancer patients. These dimensions may warrant attention in comprehensive psychological assessment and supportive care.

## 1 Introduction

Breast cancer is the most common malignant tumor among women worldwide and is the leading cause of cancer death in women across 112 countries (1). GLOBOCAN 2022 estimates, published in 2024, reported approximately 2.3 million new cases of breast cancer worldwide in 2022, and the global burden is projected to increase further by 2050 (2). In China, approximately 357,200 new cases and 75,000 deaths from breast cancer were reported among women in 2022, accounting for 15.59% and 7.94% of all new cancer cases and cancer deaths among women, respectively (3). With improvements in cancer treatment and access to care, the five-year survival rate for breast cancer patients in China was 80.9% during 2019–2021 (4). Despite advances in treatment and survival, breast cancer can still impose a considerable psychological burden (5). The diagnosis and treatment of breast cancer are often accompanied by physical and functional changes, treatment-related adverse effects, reduced quality of life, and disruptions to social relationships (6). Anxiety and depression are among the most commonly reported forms of psychological distress in breast cancer patients (7) and have been associated with poorer quality of life, lower treatment adherence, and adverse prognostic outcomes (8).

At the same time, cancer survivors may also experience positive psychological changes, including benefit finding (BF) and posttraumatic growth (PTG). Although related, BF and PTG are conceptually distinct. BF refers to perceived benefits or positive meaning derived from the cancer experience (9), whereas PTG refers to broader positive changes that emerge through the struggle with trauma, including changes in life perspective, interpersonal relationships, and self-perception (10, 11). Previous research has shown that BF and PTG are relatively common among cancer survivors, with 66.0% reporting moderate-to-high BF and 20.5% reporting moderate-to-high PTG (12). Beyond their prevalence, BF and PTG are clinically important because they are closely related to psychosocial adaptation and psychological outcomes. In breast cancer patients, BF has been positively associated with active coping and negatively associated with depressive symptoms (13). Longitudinal evidence further suggests that BF during the first year after breast cancer diagnosis predicts better psychological adjustment several years later, including lower distress and depressive symptoms (14). PTG has also been associated with psychosocial resources and outcomes. A meta-analysis reported a positive association between PTG and social support among breast cancer patients (15), and a systematic review of adult cancer survivors found that PTG was generally positively associated with health-related quality of life (16). BF, PTG, and well-being have also been examined concurrently in long-term breast cancer survivors, further underscoring the relevance of positive psychological changes to survivorship adaptation (17). Taken together, these findings suggest that anxiety, depression, BF, and PTG may represent interrelated aspects of psychological adaptation rather than isolated psychological phenomena.

In recent years, positive psychology has provided a complementary perspective for understanding psychological adaptation in cancer patients. Within this field, Martin Seligman’s PERMA framework conceptualizes well-being in terms of five components: positive emotion, engagement, relationships, meaning, and accomplishment (18, 19). Empirical work has further examined PERMA as a multidimensional model of psychological well-being, supporting its value for understanding positive psychological functioning (20). Rather than focusing only on psychopathology, this perspective emphasizes the role of internal resources and positive functioning in human adaptation (20, 21).

Drawing on the PERMA model’s emphasis on positive psychological functioning, the present study placed anxiety, depression, benefit finding, and posttraumatic growth within the same research framework to examine how distress-related dimensions and positive psychological constructs are interrelated during breast cancer adaptation.

Previous studies of these constructs have often relied on conventional variable-centered approaches, such as correlation, regression, and mediation or moderation models (13, 14, 22). Although useful for testing prespecified relationships, these approaches do not directly characterize the conditional association structure among multiple psychological dimensions simultaneously. Network analysis offers a complementary multivariate approach for estimating conditional associations among multiple psychological dimensions simultaneously (23). In recent years, network analysis has been increasingly applied in psychology and psychiatry (24, 25). Network analysis provides a novel approach by conceptualizing psychological constructs as interactive systems. In the network structure, nodes represent psychological symptoms or dimensions, whereas edges represent statistical associations between nodes after controlling for other variables in the network (26). To our knowledge, no previous study has used network analysis to examine the interrelationships among anxiety, depression, BF, and PTG in breast cancer patients. Therefore, guided by the PERMA model’s emphasis on positive psychological functioning, this study employed network analysis with the primary objectives of: (1) describing the network structure among anxiety, depression, benefit finding, and posttraumatic growth dimensions; (2) identifying central dimensions with relatively broad connections in the network; and (3) identifying bridge dimensions that connect different psychological dimension communities and provide a basis for hypotheses in future longitudinal and intervention studies.

## 2 Methods

### 2.1 Participants

This cross-sectional study was conducted between 25 September 2025 and 10 January 2026 among breast cancer patients admitted to five tertiary hospitals in northern Anhui Province, China. Participants were recruited using convenience sampling. A total of 740 questionnaires were distributed, of which 707 valid questionnaires were included in the final analysis, yielding a valid response rate of 95.5%. The inclusion criteria for this study were: 1) Pathological diagnosis of breast cancer; 2) Age ≥ 18 years; 3) No history of other malignant tumors; 4) Clear consciousness and able to communicate effectively, with no communication barriers. 5) Inpatient during the study period. Exclusion criteria included: 1) Recent use of sedative medication or the presence of metastatic cancer; 2) Concurrent severe cardiac, hepatic, or renal dysfunction; 3) Impaired hearing or speech ability preventing participation in the study. This study strictly adhered to the ethical requirements of the Declaration of Helsinki and followed applicable national guidelines. This study was approved by the Ethics Committee of Bengbu Medical University (No: Byycx25051), and all participants provided written informed consent.

### 2.2 Data collection methods

Prior to completing the questionnaire, trained members of the research team provided participants with standardized instructions, explaining the objectives, significance, and completion requirements of the study. All participants were informed that participation was entirely voluntary, that they could refuse to answer any question, and that they could withdraw from the study at any time without any adverse consequences. Inpatients completed the questionnaire on site, and the completed forms were collected immediately. An on-site review was conducted to identify any missing responses; if any omitted items were found, participants were gently reminded to complete them only if they were willing to do so, and no participant was forced to answer. Following the completion of questionnaire collection, a double-checking process was carried out by two researchers. This process strictly adhered to the inclusion and exclusion criteria, and appropriate follow-up questions were posed to clarify any ambiguous statements provided by patients. Once all questionnaires had been collected, a double-checking process was carried out by two individuals to exclude incomplete or invalid responses, thereby ensuring the authenticity and accuracy of the research data.

### 2.3 Measures

#### 2.3.1 Basic information form

The Basic Information Form was used to collect demographic and clinical characteristics for sample description, including age, marital status, occupation, educational attainment, place of residence, financial status, average nightly sleep duration, and cancer stage.

#### 2.3.2 Benefit Finding Scale

This study utilized the Chinese version of the Benefit Finding Scale, adapted by Weaver et al. (27) and translated by Liu et al. (28). The scale comprises 22 items across six dimensions: acceptance, family relations, worldview, personal growth, social relations, and health awareness. The scale employs a five-point Likert scale (1 = “not at all”, 5 = “very much”). The total score ranges from 22 to 110 points, with higher scores indicating that the individual perceives more benefits. According to previous research criteria, total scores can be classified as low (22–48 points), moderate (49–77 points), and high (≥78 points) (29). In this study, the Cronbach’s α coefficient for this scale was 0.898, indicating good internal consistency.

#### 2.3.3 Posttraumatic Growth Inventory

This tool was originally proposed by Tedeschi and Calhoun (1996) (30) and subsequently adapted by the Chinese scholar Wang Ji (31). It comprises five dimensions: appreciation of life, personal strength, new possibilities, relating to others, and spiritual change, and consists of 20 items in total. It employs a 6-point Likert scale, ranging from “not at all” to “very much”, with scores ranging from 0 to 5. Based on the total score, an individual’s level of posttraumatic growth can be categorized into three levels: a score below 60 indicates a low level of growth, 60–65 indicates a moderate level, and 66 or above indicates a high level (11). In this study, the Cronbach’s α coefficient for this scale was 0.882.

#### 2.3.4 Hospital Anxiety and Depression Scale

The Hospital Anxiety and Depression Scale was developed by Zigmond et al. in 1983 (32) and is a self-report questionnaire used to screen for anxiety and depression in hospitalized patients. The HADS comprises 14 items divided into two subscales: anxiety and depression, with each subscale containing seven items. Each item is scored using a 4-point Likert scale, ranging from 0 to 3, with higher scores indicating more severe symptoms. According to this scoring method, a score exceeding 7 indicates the presence of anxiety or depressive symptoms. In this study, the Cronbach’s α coefficient for this scale was found to be 0.872.

### 2.4 Statistical methods

For continuous variables, data are presented as means accompanied by standard deviations; for categorical variables, data are presented as frequencies accompanied by corresponding percentages. Network analysis was performed using R software (version 4.5.1). All statistical tests were conducted at a significance level of α = 0.05 (P < 0.05).

#### 2.4.1 Network analysis

##### 2.4.1.1 Network estimation

Following the methodology of Epskamp et al.(2018), network analysis was performed using the “qgraph” package in R (33, 34). To align with the study aim of examining broader construct-level relationships and to reduce model complexity, questionnaire dimension scores rather than individual items were used as nodes. Thus, each node represented a specific questionnaire dimension, and edges represented associations between dimensions. Blue edges indicated positive conditional associations, whereas red edges indicated negative conditional associations. To estimate the conditional associations between dimensions, a Gaussian Graphical Model (GGM) suitable for continuous data was employed (35). The Extended Bayesian Information Criterion Graphical Lasso (EBICglasso) algorithm was applied, with the tuning parameter set to 0.5 following Epskamp et al. (2018), a commonly used setting in psychological network analysis (33). This approach enhances the identification of true network connections while reducing weaker associations. The predictability of each node was assessed using the “mgm” package, which quantifies the proportion of a node’s variance that can be explained by its connections within the network (36). The “qgraph” package was used to optimize the visualization of the network (34).

##### 2.4.1.2 Estimation of centrality and bridge centrality

Expected influence (EI) and bridge expected influence (BEI) were calculated for each node using the R packages “qgraph” and “networktools” to assess centrality and bridge centrality, respectively (34, 37, 38). EI was selected because it retains the signs of edge weights and is therefore suitable for networks containing both positive and negative edges (39–41). Higher EI values indicate stronger overall signed connectivity with other nodes in the network (41). BEI reflects a node’s connectivity with nodes in other predefined communities. Higher BEI values indicate relatively stronger cross-community connectivity (37). In this study, the top 20% of nodes with the highest BEI values were identified as key bridge dimensions.

##### 2.4.1.3 Evaluation of network stability and accuracy

The “bootnet” package was used to evaluate network stability and accuracy (33, 42). A non-parametric bootstrap with 1,000 samples was used to estimate 95% confidence intervals (CIs) for edge weights and to perform bootstrapped difference tests for edge weights and centrality indices. Narrower 95% CIs indicate greater precision of edge-weight estimates (43). The stability of node centrality indices was assessed using a case-dropping bootstrap procedure, which reflects the maximum acceptable proportion of data that can be discarded without compromising stability. The corresponding correlation stability (CS) coefficient was calculated. A CS coefficient of ≥0.25 is considered acceptable, while values above 0.5 are preferred (33).

## 3 Results

### 3.1 Study sample

A total of 707 complete and valid questionnaires were obtained and included in the final analysis. The basic characteristics of the breast cancer patients included in this study were as follows. The mean age of the participants was 54.47 ± 11.20 years. The majority of patients were married (n=648, 91.65%), and the majority had a monthly income of less than 5,000 RMB (n=557, 78.78%); over half of the participants were in stage III cancer (n=484, 68.46%). Screening results showed that 271 breast cancer patients (38.33%, 95% CI: 34.82–41.97%) reported symptoms of anxiety (HADS1 total score > 7), and 290 participants (41.02%, 95% CI: 37.45–44.68%) reported symptoms of depression (HADS2 total score > 7); 311 participants (43.99%, 95% CI: 40.37%–47.67%) reported low levels of benefit finding (BFS total score 22–48); while 40 participants (5.66%, 95% CI: 4.18%–7.61%) demonstrated high levels of benefit finding (BFS total score ≥78); regarding “posttraumatic growth”, 470 participants (66.48%, 95% CI: 62.92%–69.86%) were at a low level (PTGI total score < 60), while 130 participants (18.39%, 95% CI: 15.71%–21.41%) reported high levels of growth (PTGI total score ≥ 66) (**Table 1**).

**Table 1:** The Sociodemographic information of the participants.

Variables	M(SD)or n%
Age	54.47 (11.20)
Marital status	
married	648 (91.65)
unmarried	59 (8.35)
Occupation	
low-skilled professions (waiters, drivers)	496 (70.16)
high-skilled professions (university teacher)	211 (29.84)
Educational level	
≤6 years	367 (51.91)
>6years	340 (48.09)
Monthly income (RMB)	
≤2000	313 (44.27)
>2000 to <5000	244 (34.51)
≥5000	150 (21.22)
Residence	
village	397 (56.15)
city	310 (43.85)
Hours of sleep per night	
≥6h	378 (53.47)
<6h	329 (46.53)
Cancer staging	
stage I	158 (22.35)
stage II	65 (9.19)
stage III	484 (68.46)
HADS-anxiety total score	6.3 ± 3.87
≤7	436 (61.67)
>7	271 (38.33)
HADS-depression total score	6.58 ± 3.89
≤7	417 (58.98)
>7	290 (41.02)
BFS total score	53.33 ± 14.07
22-48	311 (43.99)
49-77	356 (50.35)
≥78	40 (5.66)
PTGI total score	54.07 ± 13.21
<60	470 (66.48)
60-65	107 (15.13)
≥66	130 (18.39)

HADS, Hospital Anxiety and Depression Scale; BFS, Benefit Finding Scale; PTGI, Posttraumatic Growth Inventory.

### 3.2 Network structure

The results of the network analysis are shown in **Figure 1**. The network comprised 13 nodes, each representing a questionnaire dimension. Of the 78 possible edges (13 × 12/2), 71 have non-zero weights, and the network density is 0.910 (71/78). This relatively high network density indicates broad interrelations among the psychological dimensions included in this study. The bootstrap difference test for edge weights (**Supplementary Figure 1**) indicated that stronger associations were primarily observed within the same community. The strongest edge was observed between HADS1 and HADS2 (“anxiety”–”depression”, edge weight = 0.527), followed by the edge BFS1 – BFS4 (“acceptance”–”personal growth”, edge weight = 0.182) and PTGI1–PTGI3 (“appreciation of life” –”new possibilities”, edge weight = 0.135).

**Figure 1:**
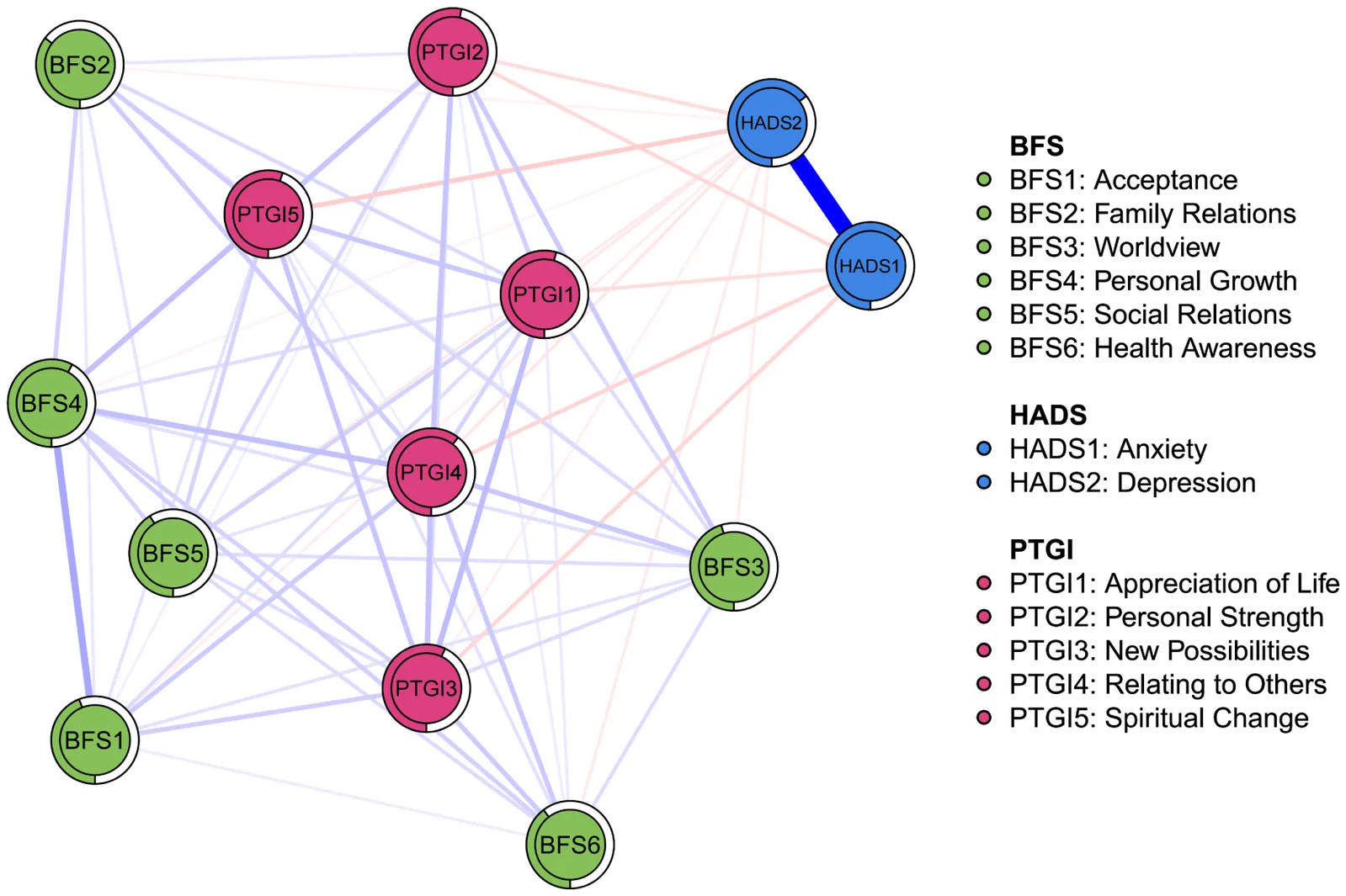
Network of benefit finding, posttraumatic growth, anxiety, and depression in breast cancer patients.

### 3.3 Network centrality and bridge centrality

The centrality indices for each node are shown in **Table 2**. Based on the expected influence (EI) values, “personal growth” (BFS4) is the most central node (node EI = 1.034), followed by “relating to others” (PTGI4, node EI = 0.846) and “spiritual change” (PTGI5, node EI = 0.781) (**Supplementary Figure 2**). Among the bridging indicators, “personal growth” (BFS4, node BEI = 0.504), “relating to others” (PTGI4, node BEI = 0.481), and “worldview” (BFS3, node BEI = 0.401) had the highest positive bridging expected influence values (BEI) (**Figure 2**), suggesting that these nodes may play a key role in connecting different communities. In contrast, anxiety (HADS1, node BEI = -0.320) and depression (HADS2, node BEI = -0.516) had negative BEI values, indicating that they were generally negatively associated with nodes in other communities. It is worth noting that “personal growth” (BFS4) and “relating to others” (PTGI4) rank highly in both the expected influence and bridge expected influence metrics, suggesting that these two nodes not only occupy central positions in the network structure but may also play bridging roles in connecting different psychological communities. A test of differences (**Supplementary Figure 3**) shows that the differences in EI values between most nodes are statistically significant.

**Table 2:** Descriptive statistics of measurement dimensions.

Node	Dimension	Mean	SD	EI	BEI
HADS1	Anxiety	0.8998	0.5533	0.2066	-0.3199
HADS2	Depression	0.9402	0.5564	0.0105	-0.5160
BFS1	Acceptance	2.4008	0.8947	0.6592	0.3286
BFS2	Family Relations	2.4342	1.0267	0.5555	0.3086
BFS3	Worldview	2.4424	0.8022	0.7134	0.4012
BFS4	Personal Growth	2.4239	0.7418	1.0344	0.5038
BFS5	Social Relations	2.4366	0.8820	0.6315	0.3397
BFS6	Health Awareness	2.4050	0.8943	0.5877	0.3121
PTGI1	Appreciation of Life	2.7030	0.8080	0.7347	0.3022
PTGI2	Personal Strength	2.7139	0.7914	0.6741	0.2683
PTGI3	New Possibilities	2.7076	0.7947	0.7794	0.3308
PTGI4	Relating to Others	2.7026	0.7995	0.8462	0.4814
PTGI5	Spiritual Change	2.6917	0.8065	0.7812	0.4009

**Figure 2:**
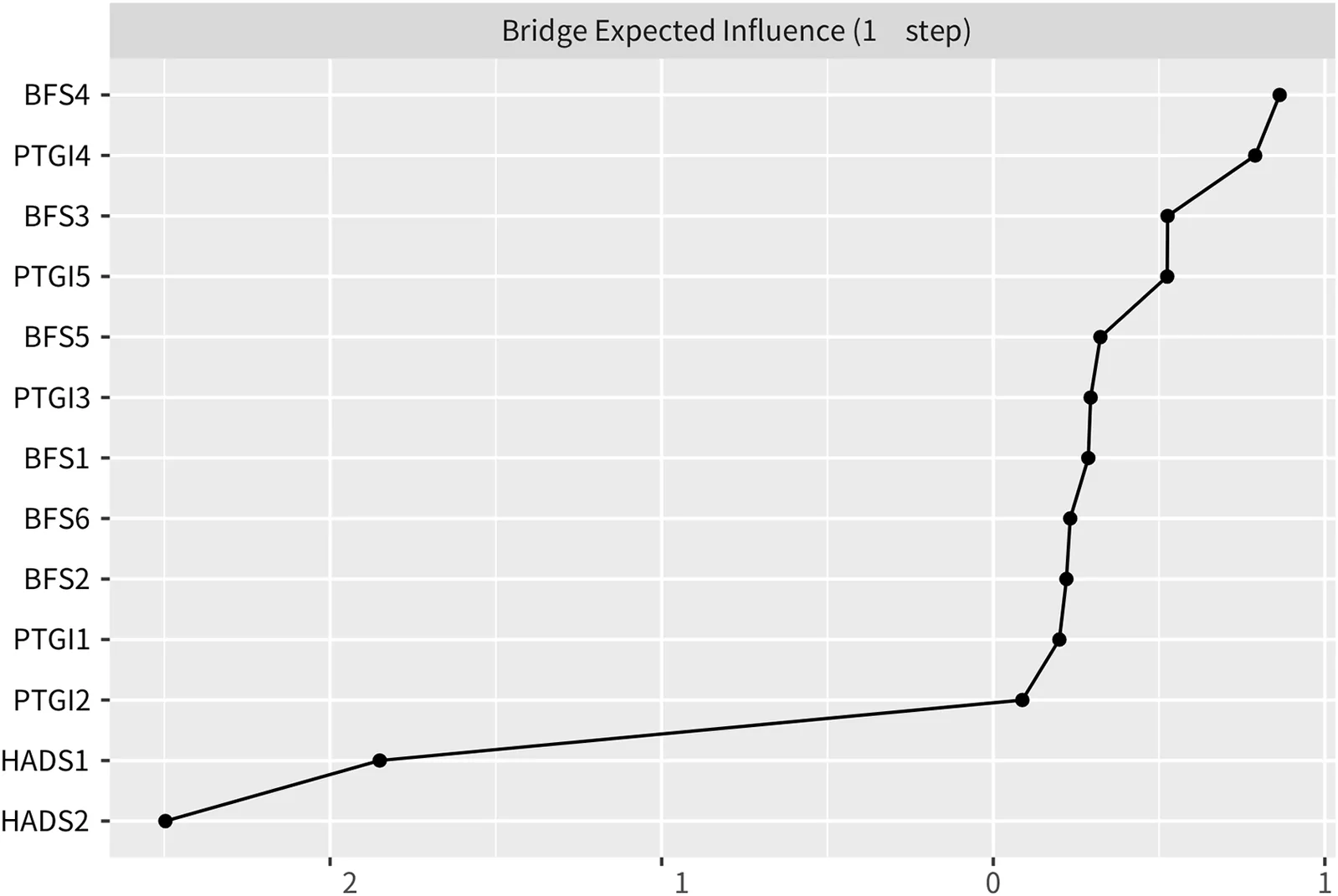
Centrality indices: bridge expected influence values.

### 3.4 Network stability and accuracy

An analysis of the stability of the EI and BEI values revealed that their CS coefficients were both 0.75 (**Supplementary Figure 4**), well above the ideal threshold of 0.5. This indicates good statistical stability of the estimated centrality indices under case-dropping bootstrap. Furthermore, the non-parametric bootstrap results showed relatively narrow 95% confidence intervals (CIs) for the edge weights, indicating relatively precise edge-weight estimates (**Figure 3**).

**Figure 3:**
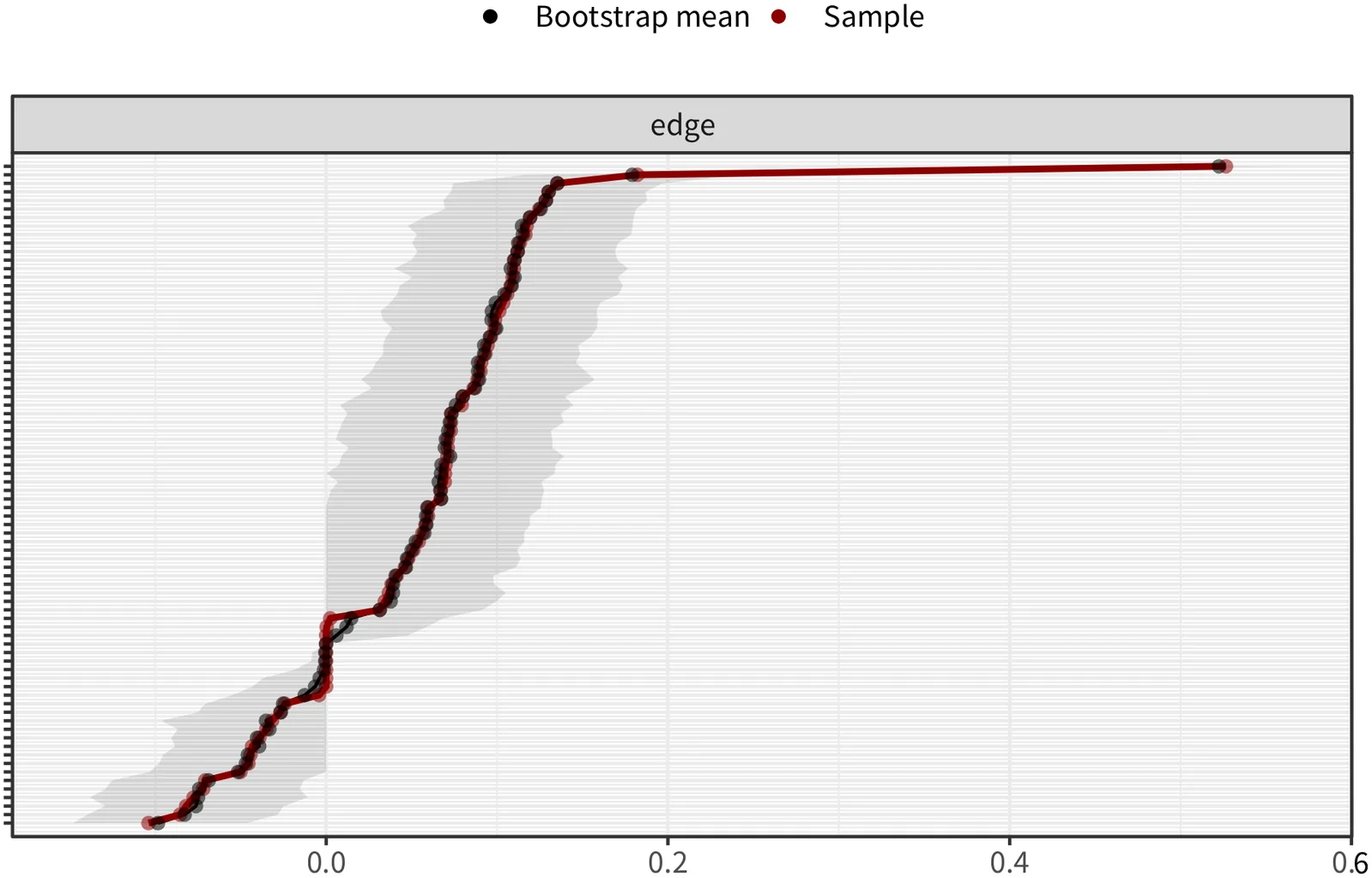
Bootstrapped confidence intervals of edge weight.

## 4 Discussion

To our knowledge, this is the first study to use network analysis to examine the interrelationships among benefit finding, posttraumatic growth, anxiety, and depression at the dimension level in a breast cancer sample. The findings highlight the central and bridge positions of “personal growth” (BFS4), “relating to others” (PTGI4), and “worldview” (BFS3), suggesting broad interrelationships between distress-related dimensions and positive psychological constructs. At the same time, the strongest association was observed between anxiety (HADS1) and depression (HADS2), both within the distress community. Although the high CS coefficients and narrow bootstrap CIs support the statistical stability of the network, they do not establish construct validity. Therefore, centrality and bridge indices should be interpreted cautiously within the present measurement framework.

### 4.1 Prevalence of anxiety and depression and their network association

The prevalence rates of anxiety symptoms (HADS1, 38.3%) and depressive symptoms (HADS2, 41.0%) in the present sample were close to the ranges reported in previous studies of breast cancer populations. Existing research has shown that the prevalence rates of anxiety symptoms among breast cancer patients are approximately 17.9%–33.3%, and that of depressive symptoms is 9.4%–66.1%, with some variation observed across different studies (44). Building on this, our study further revealed through network analysis that, among all associations in the network, the link between anxiety and depression was the strongest. This finding is consistent with previous research (45, 46); studies in cancer patients have found that there is a close association between anxiety and depressive symptoms, which manifests in the network as a highly interconnected cluster of symptoms (47). Anxiety and depression are generally considered to share common underlying psychological mechanisms, such as negative cognitive appraisals, uncertainty regarding disease prognosis, and concerns about treatment outcomes (48). However, other studies have noted that in certain populations, the direct association between anxiety and depression may be less pronounced, with symptoms such as fatigue and sleep disturbances also being involved in their broader network connections (49). This variation may be related to differences in the characteristics of the study population (such as disease stage and symptom burden) and the variables included in the network.

From a clinical perspective, the strong association between anxiety and depression underscores the importance of assessing these distress dimensions together in breast cancer patients. Comprehensive psychological assessment and supportive care that consider both anxiety and depression may provide a more complete understanding of patients’ emotional distress.

### 4.2 Central role of personal growth in the psychological network

This study found that “personal growth” (BFS4) is the most central node in the co-occurrence network. This dimension reflects positive changes in personal qualities, including greater patience, responsibility, personal strength, and coping ability (27). A prospective study of women with breast cancer also suggests that higher levels of BFS4 are associated with fewer anxiety symptoms (50). Taken together, these findings suggest that BFS4 is closely related to patients’ coping resources and emotional adjustment, which may help explain its broad connectedness and central position in the present network. This finding is consistent with the research by Kathryn E. Weaver et al., who found that “BFS4” is a key component of the benefit-finding structure in cancer patients and is closely associated with psychological adaptation (27). Furthermore, Qiu et al. found that the “BFS4” dimension had the highest score among the dimensions of benefit finding, further indicating the prominence of perceived personal growth in cancer-related positive psychological changes (51). However, some research findings do not fully align with those of this study. The prominence of BFS4 should nevertheless be interpreted in relation to the constructs and dimensions included in a given study. For example, a Turkish study examined posttraumatic growth in relation to resilience and quality of life among breast cancer patients rather than benefit-finding dimensions (52). Differences in the constructs and measurement frameworks considered across studies may partly influence which positive psychological dimensions appear most prominent.

From a clinical perspective, the central position of BFS4 suggests that personal growth-related psychological resources deserve greater attention in the psychological care of breast cancer patients. Previous longitudinal research showed that self-efficacy predicted personal growth after cancer surgery, with assimilative coping also being associated with subsequent personal growth (53). Furthermore, cognitive behavioral stress management has been shown to facilitate changes in the personal growth dimension of benefit finding among women with breast cancer (54). Therefore, strategies aimed at strengthening coping skills and confidence in managing cancer-related challenges may represent promising directions for future intervention research, particularly among patients with lower levels of personal growth.

### 4.3 Bridging role of positive psychological dimensions

Bridge centrality analysis further identified “personal growth” (BFS4), “relating to others” (PTGI4), and “worldview” (BFS3) as key bridge dimensions in the network. Notably, BFS4 and PTGI4 ranked among the top dimensions in both expected influence and bridge expected influence. Their bridge positions suggest relatively close connections with dimensions across different psychological communities and may help characterize the interrelationships between distress-related dimensions and positive psychological constructs.

As discussed above, the bridge position of BFS4 may be related to the close associations of personal growth with coping-related resources and emotional adjustment. PTGI4 (“relating to others”) reflects positive changes in interpersonal connectedness, particularly greater closeness to others and greater acceptance of relying on and needing others (30, 31). This interpretation is consistent with Romeo et al., who reported a positive association between perceived social support and the “relating to others” dimension of posttraumatic growth in breast cancer survivors (55). BFS3 (“worldview”) encompasses perceived changes concerning purpose in life, the human need for love, religious faith, and concern for the future of humankind, reflecting broader shifts in patients’ worldview following the cancer experience (27, 28). Such shifts in worldview may also relate to how patients interpret their cancer experience and perceive positive changes during psychological adaptation. Previous studies have also documented associations between positive psychological constructs and psychological distress. Urcuyo et al. (2005) reported that benefit finding was negatively associated with depressive symptoms (56), while Aliche et al. found that anxiety was positively associated with depression and negatively associated with posttraumatic growth (57). The present study extends this evidence by identifying specific positive psychological dimensions that show relatively broad connections across different psychological communities.

From a clinical perspective, the bridge positions of BFS4, PTGI4, and BFS3 highlight the potential importance of personal growth-related resources, interpersonal relationships, and cognitive perspectives in the psychological care of breast cancer patients. Considering these domains alongside emotional distress in comprehensive psychological assessment and supportive care may provide a more integrated approach to understanding and supporting patients’ psychological adaptation.

### 4.4 Integrating the findings from a PERMA perspective

From a broader positive psychology perspective, the present findings can be contextualized within the PERMA framework. PERMA emphasizes multidimensional positive psychological functioning rather than defining mental health solely by the absence of psychological distress (18–20). Previous research has distinguished PERMA-related well-being domains from ill-being dimensions such as anxiety and depression, while showing that they remain interrelated (20). Network-based PERMA studies have further highlighted the interconnections among well-being components and conceptualized positive functioning as a network of interconnected elements (58, 59). Accordingly, the central or bridge positions of positive psychological dimensions such as personal growth, relating to others, and worldview in the present network may be understood in terms of the interrelationship between positive psychological functioning and psychological distress.

Previous studies in breast cancer populations further support the clinical relevance of a PERMA-informed positive psychology perspective. A systematic review and meta-analysis showed beneficial effects of PERMA-based positive psychological interventions on psychological outcomes in cancer patients (60). More recently, Wang et al. reported that an intervention using PERMA as a framework was more effective in reducing subthreshold depression and improving psychosocial adjustment among patients with newly diagnosed breast cancer (61). These findings support the clinical value of considering positive psychological functioning alongside psychological distress in breast cancer care. Overall, PERMA may serve as a broad conceptual perspective for integrating the present findings and providing a more comprehensive understanding of psychological adaptation in breast cancer patients.

### 4.5 Limitations

This study has several limitations. First, the cross-sectional design precludes conclusions about causal relationships among the variables. Second, the network nodes were based on questionnaire dimensions rather than individual items or symptoms, which may have obscured more detailed associations and contributed to the relatively high network density. In addition, treatment modality, time since diagnosis, recurrence status, and cancer stage were not incorporated as clinical covariates in the network analysis, precluding examination of their potential influence on the findings. Third, all data were derived from self-report questionnaires, which may introduce reporting bias and limit the clinical interpretation of the findings. Fourth, the sample was limited to breast cancer inpatients from tertiary hospitals in northern Anhui Province, China, and differences in cultural and social backgrounds may limit the generalizability of the findings. Future studies should adopt multicenter, large-sample longitudinal designs, conduct sensitivity analyses using stronger regularization methods, and incorporate relevant clinical covariates, clinical assessments, or structured interviews to enhance the clinical validity of the findings, thereby further validating the present findings and providing a basis for developing more scientifically grounded and appropriate interventions to promote overall psychological adaptation and recovery among breast cancer patients.

## 5 Conclusion

BFS4 (“personal growth”) emerged as the most central dimension in the network, highlighting its importance within the interrelationships among benefit finding, posttraumatic growth, anxiety, and depression. Furthermore, BFS4, PTGI4 (“relating to others”), and BFS3 (“worldview”) showed important bridge positions, suggesting close connections between positive psychological constructs and distress-related dimensions. These findings highlight the potential relevance of personal growth, interpersonal relationships, and worldview in the psychological care of breast cancer patients and support considering these positive psychological dimensions alongside anxiety and depression in comprehensive psychological assessment and supportive care.

## Data Availability

The raw data supporting the conclusions of this article will be made available by the authors, without undue reservation.
