# Supplementary material for: A network analysis of benefit finding, posttraumatic growth, anxiety, and depression in breast cancer patients
Source: Front Oncol. 2026 Sep 17;16:1845985. doi: 10.3389/fonc.2026.1845985 (PMC13627068; doi:10.3389/fonc.2026.1845985)
Supplement: Supplementary file 1 [file DataSheet1.docx]

Supplemental file

Figure S1. Estimation of edge weight difference by bootstrapped difference test.

The black boxes represent an edge that differs significantly from another edge (α = 0.05).

Figure S2. Centrality indices: expected influence values.

Figure S3. Nonparametric bootstrapped difference test

The black boxes represent a node that differs significantly from another node (α = 0.05

Figure S4. The stability of centrality and bridge centrality indices according to a case-dropping bootstrap method.

The figure shows the average correlations between centrality indices of the original sample and subsamples with fewer participants.
